# Metabolic changes with intermittent fasting

**DOI:** 10.1111/jhn.13253

**Published:** 2024-01-10

**Authors:** Maria G. Lange, Alice A. Coffey, Paul C. Coleman, Thomas M. Barber, Thijs Van Rens, Oyinlola Oyebode, Sally Abbott, Petra Hanson

**Affiliations:** ^1^ Warwick Medical School University of Warwick Coventry UK; ^2^ Warwickshire Institute for the Study of Diabetes Endocrinology and Metabolism University Hospitals Coventry and Warwickshire Coventry UK; ^3^ Department of Economics University of Warwick Coventry UK; ^4^ Wolfson Institute of Population Health Queen Mary University of London London UK; ^5^ Department of Dietetics University Hospitals Coventry and Warwickshire NHS Trust Coventry UK; ^6^ Research Centre for Intelligent Healthcare Coventry University Coventry UK

**Keywords:** alternate day fasting, intermittent fasting, obesity, weight loss

## Abstract

**Background:**

The prevalence of obesity is rising globally and effective strategies to treat obesity are needed. Intermittent fasting, a dietary intervention for weight management, has received growing interest from the general public, as well as healthcare professionals, as a form of lifestyle intervention.

**Methods:**

We executed a rapid review using PUBMED database to identify systematic reviews that examined the impact of intermittent fasting on metabolic indices, published between 2011 and 2022.

**Results:**

Intermittent fasting leads to weight loss of a similar magnitude to continuous energy restriction. Most of the evidence shows that intermittent fasting leads to greater fat loss as measured by fat mass (kg) or body fat percentage compared to an *ad libitum* diet, but fat loss attained during intermittent fasting is not significantly different to continuous energy restriction, although recent evidence shows intermittent fasting to be superior. There is mixed evidence for the impact of intermittent fasting on insulin resistance, fasting glucose and lipid profile. Some studies focused on populations of Muslim people, which showed that Ramadan fasting may lead to weight loss and improvement of metabolic parameters during fasting, although the effects are reversed when fasting is finished.

**Conclusions:**

Intermittent fasting is more effective than an *ad libitum* dietary intake, and equally or more effective as continuous energy restriction, for weight management. However, there is inconclusive evidence on whether intermittent fasting has a clinically beneficial effect on glucose and lipid metabolism.

## Key points

- Intermittent fasting (IF) encompasses time restricted eating, alternate day fasting and intermittent energy restriction
- IF is more effective for weight loss and reduction with respect to body mass index than *ad libitum* dietary intake
- IF is equally or more effective as continuous energy restriction for weight management

## Graphical abstract

A rapid review of systematic reviews examining the impact of intermittent fasting (IF) on metabolic indices. IF is more effective than an *ad libitum* dietary intake, and equally or more effective as continuous energy restriction, for weight management. The evidence on IF impact on glucose and lipid metabolism is inconclusive.

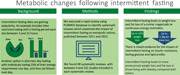

## INTRODUCTION

Globally, between 1980 and 2019, the prevalence of obesity increased from 3.2% to 12.2% in men and from 6% to 15.7% in women.^1^ Obesity can lead to various adverse health outcomes, including type 2 diabetes (T2D), heart disease and some cancers, highlighting the importance of weight management. Treating obesity is likely to be cost‐effective, reducing the prevalence of associated diseases in the future.^2^ Until recent years, dietary interventions for weight loss have focused on continuous energy restriction (CER), with clinical guidelines recommending that people living with obesity should aim for a daily energy intake that is 600 kcal less than their total energy expenditure.^3^ Although this is an effective strategy for weight loss, adherence to CER has been reported to be as low as 32%.^4^, ^5^ Consequently, weight maintenance is rarely sustainable in the long term. Therefore, alternative weight loss strategies are needed to provide person‐centred approaches.

In recent years, intermittent fasting (IF) has been subject to growing research interest. Although definitions vary slightly, the concept of IF is energy restriction through abstaining from food for a specified amount of time, rather than CER over the course of a whole day.^6^ There are several variations of IF, including:

− **Time restricted eating** (TRE): this is a fasting period of between 3 and 21 h per day (although most commonly >12 h). Ramadan diurnal intermittent fasting (RDIF) is the fasting period during Ramadan, which is practised by Muslim people from sunrise to sunset on consecutive days over a 1‐month period and therefore aligns with TRE. Energy restriction is not required.
− **Alternate day fasting** (ADF): the individual restricts their energy consumption to 25% of their daily energy requirements on 1 day, and then an *ad libitum* diet is followed the next day, on a repeating basis. Alternatively, a 24‐h fast period for 1 day is followed by an *ad libitum* diet the next day, also known as periodic fasting.
− **Intermittent energy restriction** (IER): This is a less defined duration of fasting, where the individual undertakes periods of energy restriction alternated with periods of habitual intake or minimally restricted dietary intake.

### Rationale

The previous literature has suggested that IF may be a more sustainable approach to weight loss because the duration of restriction is not as prolonged.^7^ As the popularity of IF is increasing and interest is growing, the effectiveness of IF needs to be appraised to provide evidence‐based sustainable weight loss recommendations. Hence, our aim was to synthesise the available evidence on the metabolic effects of IF from the findings of published, systematic reviews.

## METHODS

We conducted a rapid overview (an umbrella review) of systematic reviews published in the medical literature on the impact of IF on metabolic effects. When compared with full reviews, rapid reviews incur no discernible impacts on derived conclusions.^8^ Rapid reviews summarise the highest level of academic evidence and provide healthcare professionals with a clear summary of existing evidence.^9^ PUBMED was searched by one researcher (ML) to identify relevant literature published between November 2011 and November 2022. Studies were included if they were systematic reviews written in English language. Systematic reviews were chosen as they are more robust than narrative reviews, with clearly defined primary and secondary objectives, explicit methods of extraction and synthesis of the data. We included any systematic review that observed the effects of any IF dietary intervention compared to a control group, as measured by changes in body composition or metabolic indices. Body composition could include body mass index (BMI) (kg/m^2^), weight (kg), body fat mass (kg) or fat mass as a proportion of total body weight (%). Diabetes control was measured by fasting glucose (mg/dl) and HbA1c (mmol/mol). Insulin resistance was measured by fasting insulin (mIU/ml) and HOMA‐IR. Changes in lipids were measured by total cholesterol (TC) (mg/dl), triglycerides (TG) (mg/dl), low‐density lipoprotein‐cholesterol (LDL‐C) (mg/dl) and high‐density lipoprotein‐cholesterol (HDL‐C) (mg/dl). Data were collected using a pre‐defined framework and included the surname of first authors, publication year, study design, sample size, analysis, population, intervention/exposure and outcomes.

## RESULTS

This review identified 48 systematic reviews (Table 1). The number of included studies in each systematic review ranged from 4 to 101. The individual studies included a combination of interventional and observational studies. Nine of these reviews specifically examined the effects of fasting during Ramadan, whereas the remaining studies examined a range of conventional IF approaches. The study populations were diverse, including predominantly adults with overweight (≥25 kg/m^2^) or obesity (≥30 kg/m^2^), with and without associated metabolic comorbidities. The majority of populations included in Ramadan studies were healthy adults. The control diets that were used within the included systematic reviews were either CER or an *ad libitum* diet (consuming a normal diet which is non‐restrictive).

**Table 1:** Included systematic reviews.

Main author	References	Title	Date range	Study designs included	Population	Intervention (duration)	Control	Number of studies
R. Adafer	[10]	Food Timing, Circadian Rhythm and Chrononutrition: A Systematic Review of Time‐Restricted Eating's Effects on Human Health	2014−2020	RCT, non‐randomised clinical trials, single‐arm	Adults with overweight and obese. Some with T2D/pre‐diabetes.	TRE–eTRE, dTRE (4 days to 4 months)	Standard diet, extended feeding window	22
B. Alhamdan	[31]	Alternate‐day versus daily energy restriction diets: which is more effective for weight loss? A systematic review and meta‐analysis	2000–2015	Experimental studies	Adults with obesity	ADF (3 to 12 weeks)	VLCD	28
M. Allaf	[11]	Intermittent fasting for the prevention of cardiovascular disease	2011–2019	RCT	adults over 18	IER (4 weeks to 6 months)	Standard diet or CER	18
D. Ashtary‐Larky	[12]	Effects of intermittent fasting combined with resistance training on body composition: a systematic review and meta‐analysis	2010–2020	RCT	Healthy Adults performing RT, 1 study with overweight/obese	TRE, ADF, Ramadan (4 to 12 weeks)	Standard diet + RT	8
D. Bitsanis	[41]	The Effect of Early Time‐Restricted Feeding on Glyceamic Profile in Adults: A Systematic Review of Interventional Studies	2017–2020	RCTs, non‐randomised clinical trials	Adults with normal weight, overweight, obese with or without MetS	eTRE (3 days to 5 weeks)	Standard diet or dTRE	5
J. Bonnet	[13]	Breakfast Skipping, Body Composition, and Cardiometabolic Risk: A Systematic Review and Meta‐Analysis of Randomized Trials	1992–2017	RCT	adults above 18	Omitting breakfast (4 to 16 weeks)	Having breakfast	7
E. Borgundvaag	[14]	Metabolic Impact of Intermittent Fasting in Patients With Type 2 Diabetes Mellitus: A Systematic Review and Meta‐analysis of Interventional Studies	1991–2018	RCT	Adults with T2D, mostly obese	TRE, IER, CER (7 and 168 days)	Standard diet	7
J. Chen	[15]	Missing puzzle pieces of time‐restricted‐eating (TRE) as a long‐term weight‐loss strategy in overweight and obese people? A systematic review and meta‐analysis of randomized controlled trials	2016–2021	RCTs, non‐randomised clinical trials	Adults with overweight or obesity. Some with MetS	TRE (6 to 12 weeks)	Standard diet or extended eating window	12
Y. Cho	[16]	The Effectiveness of Intermittent Fasting to Reduce Body Mass Index and Glucose Metabolism: A Systematic Review and Meta‐Analysis	2011–2018	RCT	Adults over 18	IER (4 and 24 weeks)	Standard diet or CER	12
J. Choi	[32]	Effect of Carbohydrate‐Restricted Diets and Intermittent Fasting on Obesity, Type 2 Diabetes Mellitus, and Hypertension Management: Consensus Statement of the Korean Society for the Study of Obesity, Korean Diabetes Association, and Korean Society of Hypertension	2000–2021	RCT	Adults with overweight or obesity (BMI > 23 Korean Obesity Criteria)	TRE, ADF, IER (8 weeks to 24 weeks)	Standard diet or CER	8 on IF (50 on carbohydrate restricted diets)
I. Cioffi	[33]	Intermittent versus continuous energy restriction on weight loss and cardiometabolic outcomes: a systematic review and meta‐analysis of randomized controlled trials	1998–2018	RCT	Adults with overweight and obesity, some with T2D	IER (8 to 24 weeks)	CER	11
J. M. Correia	[45]	Effects of Ramadan and Non‐Ramadan Intermittent Fasting on Body	1995–2020	RCT, observational studies	adults over 18	34 studies on Ramadan, 32 TRE (4 weeks)	Standard diet	66
Y. Cui	[17]	Health Effects of Alternate‐Day Fasting in Adults: A Systematic Review and Meta‐Analysis	2013–2019	RCT	Adults with normal weight, overweight or obesity	ADF (4 to 48 weeks)	Standard diet	7
A. Ezzati	[4]	The Effects of Isocaloric Intermittent Fasting vs Daily Caloric Restriction on Weight Loss and Metabolic Risk Factors for Noncommunicable Chronic Diseases: A Systematic Review of Randomized Controlled or Comparative Trials	2000–2022	RCT	Adults with overweight or obesity. Some T2D or MetS	TRE, ADF, 4:3, 5:2 (8 to 52 weeks)	CER	13
M. Faris	[51]	A systematic review, meta‐analysis, and meta‐regression of the impact of diurnal intermittent fasting during Ramadan on glucometabolic markers in healthy subjects	1982–2020	Prospective observational studies	Healthy adults	RDIF (4 weeks)	Self‐control (pre/post RDIF)	72
H. Fernando	[46]	Effect of Ramadan Fasting on Weight and Body Composition in Healthy Non‐Athlete Adults: A Systematic Review and Meta‐Analysis	1982–2018	observational studies	Adults between 16–70 years	Fasting during Ramadan: 10–17 h (4 weeks)	return to normal diet post Ramadan	70
L. Gu	[6]	Effects of Intermittent Fasting in Human Compared to a Non‐intervention Diet and Caloric Restriction: A Meta‐Analysis of Randomized Controlled Trials	2007–2021	RCT	Adults with overweight or obesity. Some with T2D or MetS	ADF, TRE, IER, RDIF (median intervention time 3 months)	Standard diet or CER	43
L. Harris	[18]	Intermittent fasting interventions for treatment of overweight and obesity in adults: a systematic review and meta‐analysis	1989–2015	RCT and pseudo‐randomised clinical trials	Adults with overweight or obesity	ADF (3 to 12 months)	Standard diet or CER	6
M. Headland	[19]	Weight‐Loss Outcomes: A Systematic Review and Meta‐Analysis of Intermittent Energy Restriction Trials Lasting a Minimum of 6 Months	1989–2014	RCT, randomised parallel study	Adults with overweight or obesity. Some with T2DM	IER, modified ADF, 5:2, Intermittent use of CER or VLCD (8 weeks to 2 years)	CER	9
B. D Horne	[7]	Health effects of intermittent fasting: hormesis or harm? A systematic review	2008–2013	RCT, prospective observational trials	Adults without T2D	TRE (2 days to 12 weeks)	Standard diet	7
H. Jahrami	[54]	A systematic review, meta‐analysis, and meta‐regression of the impact of diurnal intermittent fasting during Ramadan on body weight in healthy subjects aged 16 years and above	1982–2019	Prospective observational study, interventional clinical studies	Healthy adults	RDIF (4 weeks)	Self‐control (pre/post RDIF)	85
H. Jahrami	[55]	Does four‐week consecutive, dawn‐to‐sunset intermittent fasting during Ramadan affect cardiometabolic risk factors in healthy adults? A systematic review, meta‐analysis, and meta‐regression	1982–2020	Observational studies, RCT, quasi‐experimental, experimental	Adults	RDIF (4 weeks)	Self‐control (pre/post RDIF)	91
H. Jahrami	[49]	The impact of Ramadan fasting on the metabolic syndrome severity in relation to ethnicity and sex: Results of a systematic review and meta‐analysis	1950–2022	Cohort studies	Healthy adults	RDIF (4 weeks)	Self‐control (pre/post RDIF)	101
J. Kang	[20]	Effect of Time‐Restricted Feeding on Anthropometric, Metabolic, and Fitness Parameters: A Systematic Review	2000–2021	RCT, non‐randomised clinical trials	Adults with normal weight, overweight or obesity	TRE (1 to 16 weeks)	Standard diet or cross‐over	23
C. Lima	[34]	Impact of intermittent fasting on body weight in overweight and obese individuals	2015–2019	RCT	Adults with overweight or obesity	IF (8 weeks to 4 months)	CER	4
L. Liu	[21]	Metabolic Efficacy of Time‐Restricted Eating in Adults: A Systematic Review and Meta‐Analysis of Randomized Controlled Trials	2016–2022	RCT	Adults with normal weight, overweight or obesity	TRE (4 weeks to 12 months)	Standard diet	17
M. Mazidi	[47]	The effect of Ramadan fasting on cardiometabolic risk factors and anthropometrics parameters: A systematic review	1982–2014	Case series, cohort studies, randomised studies	Adults	RDIF (4 weeks)	Self‐control (pre/post RDIF)	16
H. Meng	[44]	Effects of intermittent fasting and energy‐restricted diets on lipid profile: A systematic review and meta‐analysis	2006–2019	Clinical trials—cross‐over or parallel design	Adults with overweight or obesity. Some healthy some with T2D or MetS	IF or CER (4 weeks to 12 months)	Standard diet	35
P. Mirmiran	[50]	Effects of Ramadan intermittent fasting on lipid and lipoprotein parameters: An updated meta‐analysis	1978–2019	Observational studies and clinical trials	Healthy adults, pregnant women, athletic individuals	RDIF (4 weeks)	Self‐control (pre/post RDIF), non‐fasting control	33
S. Moon	[22]	Beneficial Effects of Time‐Restricted Eating on Metabolic Diseases: A Systemic Review and Meta‐Analysis	2007–2020	RCT, non‐randomised clinical trials	Healthy adults, some with MetS	TRE (25 days to 12 weeks)	Standard diet or pre/post TRE	21
E. Morales‐Suarez‐Varela	[35]	Intermittent Fasting and the Possible Benefits in Obesity, Diabetes, and Multiple Sclerosis: A Systematic Review of Randomized Clinical Trials	2015–2020	RCT and reviews	Adults with obesity and T2D	IF (2 days to 4 weeks)	Standard diet	31
J.Park	[23]	Effect of alternate‐day fasting on obesity and cardiometabolic risk: A systematic review and meta‐analysis	2013–2019	RCT	adults above 18	Alternate day fasting (4 to 24 weeks)	normal diet, CER, TRF	8
M. Pellegrini	[24]	Effects of time‐restricted feeding on body weight and metabolism. A systematic review and meta‐analysis	2003–2018	RCT, prospective and retrospective observational study	Adults without T2D	TRE: Studies in Ramadan between 12 and 19 h fasts. Other trials 16–20 h fasts. (4 weeks)	Standard diet	11
I.Pureza	[42]	Effect of early time‐restricted feeding on the metabolic profile of adults with excess weight: A systematic review with meta‐analysis	2014–2020	RCT	Adults with overweight or obesity	eTRE with or without CER (1 day to 12 weeks)	Extended eating window, dTRE, Standard diet	9
Y. Roman	[36]	Effects of intermittent versus continuous dieting on weight and body composition in obese and overweight people: a systematic review and meta‐analysis of randomized controlled trials	1990–2018	RCT	Adults with overweight or obesity. Some T2D	IF (12 to 52 weeks)	CER	9
B. Sadeghirad	[48]	Islamic fasting and weight loss: a systematic review and meta‐analysis	1982–2011	observational studies	adults above 18	RDIF (4 weeks)	return to normal diet post Ramadan	35
C. Sandoval	[40]	Effectiveness of intermittent fasting to potentiate weight loss or muscle gains in humans younger than 60 years old: a systematic review	2009–2019	RCT, cross‐sectional studies	Adults with normal weight, overweight and obesity	IER, eTRE, RDIF, TRE (not provided)	Standard diet, cross over, pre/post RDIF	10
L. Schwingshackl	[5]	Impact of intermittent energy restriction on anthropometric outcomes and intermediate disease markers in patients with overweight and obesity: systematic review and meta‐analyses	1989–2019	RCT	Adults with overweight or obesity. Most healthy, some T2D	IER, ADF (12 to 42 weeks)	Standard diet or CER	17
R. Seimon	[25]	Do intermittent diets provide physiological benefits over continuous diets for weight loss? A systematic review of clinical trials	1970–2014	RCT, pilot studies	Adults with normal weight, overweight or obesity.	IER, ADF (2 to 104 weeks)	Standard diet, CER, some no control	40
E. Thackrey	[43]	The effects of diet on weight and metabolic outcomes in patients with double diabetes: A systematic review	2013–2018	RCT, non‐randomised clinical trials, observational studies	Adults with double diabetes (T1D and IR, MetS, overweight/obesity)	IF, CER, Fasting + CER, MedDiet, low‐fat diet (7 days to 6 months)	Standard diet or different intensity of dietary exposure	6 (only 1 IF)
R. Vitale	[37]	The Effects of Intermittent Fasting on Glycemic Control and Body Composition in Adults with Obesity and Type 2 Diabetes: A Systematic Review	2000–2021	RCT	Adults with T2D and overweight or obesity	IER, 5:2, TRE, modified fasting (12 to 24 weeks)	Standard diet or CER	5
X. Wang	[38]	Intermittent fasting versus continuous energy‐restricted diet for patients with type 2 diabetes mellitus and metabolic syndrome for glycemic control: A systematic review and meta‐analysis of randomized controlled trials	1998–2020	RCT	Adults with T2D or MetS	5:2 or ADF (8 weeks to 12 months)	CER	5
X. Wei	[26]	Intermittent Energy Restriction for Weight Loss: A Systematic Review of Cardiometabolic, Inflammatory and Appetite Outcomes	2012–2020	RCT	Adults with overweight or obesity. Some T2D or MetS	IER, ADF, 5:2, TRE, 6:1, 1week on/1 off (8 to 52 weeks)	Standard diet or CER, with or without exercise	27
S. Welton	[27]	Intermittent fasting and weight loss: Systematic review	2000–2019	RCT, clinical trials with no controls	Adults with overweight or obesity, some with T2D	IF (2 to 52 weeks)	Standard diet or CER	27
Z. Xie	[28]	Effects of time‐restricted feeding with different feeding windows on metabolic health: A systematic review of human studies	2005–2021	RCT, non‐randomised trials, cohort studies	Adults with overweight or obesity, some with prediabetes/T2D	eTRE or dTRE (2 to 12 weeks)	Standard diet or CER	19
F. Yang	[29]	Effect of Epidemic Intermittent Fasting on Cardiometabolic Risk Factors: A Systematic Review and Meta‐Analysis of Randomized Controlled Trials	1998–2020	RCT	Adults	IF or IER (not provided)	Standard diet or CER	55
X. Yuan	[30]	Effect of Intermittent Fasting Diet on Glucose and Lipid Metabolism and Insulin Resistance in Patients with Impaired Glucose and Lipid Metabolism: A Systematic Review and Meta‐Analysis	2011–2020	RCT	Adults with MetS or obesity	IF (7 days to 12 months)	Pre/Post intervention	10
Q. Zhang	[39]	Intermittent Fasting versus Continuous Calorie Restriction: Which Is Better for Weight Loss?	2000–2020	RCT, pilot trials	Adults with overweight or obesity. Some T2D or MetS	IF, ADF, 5:2 (6 to 48 weeks)	CER	11

Abbreviations: ADF, alternate day fasting; CER, continuous energy restriction; IER, intermittent energy restriction; MetS, metabolic syndrome; RCT, randomised controlled trial; RDIF, Ramadan diurnal intermittent fasting; RT, resistance training; TRE, time restricted eating (eTRE early, dTRE delayed); T2D, type 2 diabetes mellitus; VLCD, very low calorie dieting.

### Effect on weight/BMI

Overall, the reviewed evidence suggests IF regimes consistently resulted in weight loss. The duration of interventions ranged from 1 to 104 weeks.

#### IF versus *ad libitum*

All reviews concluded that IF led to weight loss compared an *ad libitum* diet,^5^, ^6^, ^7^, ^10^, ^11^, ^12^, ^13^, ^14^, ^15^, ^16^, ^17^, ^18^, ^19^, ^20^, ^21^, ^22^, ^23^, ^24^, ^25^, ^26^, ^27^, ^28^, ^29^, ^30^ with one review reporting a reduction of initial body weight of up to 13%.^27^ The majority of studies were conducted in individuals with a BMI ≥ 25 kg/m^2^, with four reviews finding that IF was more effective for weight loss in those with a higher body weight initially.^14^, ^20^, ^23^, ^29^ Conversely, one study found a significant reduction in body weight in lean and overweight but not among individuals with obesity.^21^ Two studies found no change in weight in lean and metabolically healthy participants.^22^, ^25^ BMI decreased after IF compared to an *ad libitum* diet in 13 out of 14 reviews.^6^, ^11^, ^12^, ^15^, ^16^, ^17^, ^20^, ^21^, ^23^, ^24^, ^25^, ^27^, ^29^, ^30^

#### IF versus CER

Nineteen reviews found a comparable weight loss following IF and CER.^4^, ^5^, ^6^, ^11^, ^18^, ^19^, ^23^, ^25^, ^26^, ^27^, ^31^, ^32^, ^33^, ^34^, ^35^, ^36^, ^37^, ^38^, ^39^ In terms of BMI change, five reviews compared IF to CER, finding no significant difference between the two interventions.^11^, ^25^, ^27^, ^38^, ^39^

### Fat mass

Changes in fat mass following IF compared to control diets (CER or *ad libitum*) varied between reviews, with the majority of studies supporting that IF significantly reduces fat loss compared to an *ad libitum* diet, but no difference between IF and CER diets.

#### IF versus *ad libitum*

Seventeen reviews reported the effects of IF on fat mass compared to an *ad libitum* diet. The study populations were highly heterogenous across various BMI categories. Some studies included healthy adults, whereas others included adults with metabolic diseases. Thirteen reviews stated a significant reduction in fat mass (ranging from 0.46 kg and 3.24 kg).^6^, ^7^, ^10^, ^12^, ^15^, ^17^, ^18^, ^20^, ^21^, ^22^, ^23^, ^26^, ^29^ One meta‐analysis showed a directional reduction in reduced FM in IF but this was not statistically significant (*p* = 0.151).^16^ The remaining three studies showed inconclusive results,^13^, ^24^, ^40^ although, no studies found IF to increase fat mass.

#### IF versus CER

Nine reviews reported similar fat mass loss between IF and CER.^4^, ^25^, ^26^, ^27^, ^32^, ^33^, ^36^, ^37^, ^39^ Three meta‐analyses of interventional trials (randomised and non‐randomised) stated that IF results in higher levels of fat mass loss than CER, with a mean difference range from −0.66 kg to −3.31 kg.^5^, ^18^, ^31^ These three reviews predominantly investigated the ADF form of IF, whereas the other nine reviews included various IF types. Further studies are needed to determine whether ADF is superior to other forms of IF with respect to achieving greater levels of fat mass loss than CER.

### Insulin resistance and diabetes control

Twenty‐eight reviews reported changes in fasting glucose, insulin, HbA1c and insulin resistance, with mixed results.

#### IF versus *ad libitum*

When compared with an *ad libitum* diet, 10 reviews found fasting glucose to be significantly reduced during IF,^10^, ^16^, ^21^, ^22^, ^24^, ^29^, ^30^, ^40^, ^41^, ^42^ whereas nine reviews found that there was no significant difference compared to an *ad libitum* diet.^5^, ^6^, ^13^, ^17^, ^20^, ^25^, ^26^, ^28^, ^37^ All seven reviews that reported HbA1c levels found no significant difference between IF and control groups.^5^, ^14^, ^21^, ^25^, ^29^, ^30^, ^37^ Four of these reviews did not include any participants with T2D and therefore most participants did not have any glucose abnormalities at baseline.^30^ Two out of three reviews that included participants with T2D showed a trend towards decreasing HbA1c that did not meet statistical significance.^14^, ^37^

Twelve reviews analysed fasting insulin levels in IF versus an *ad libitum* diet. The number of reviews that identified no difference^13^, ^20^, ^24^, ^26^, ^28^, ^42^ was equal to the number of reviews that identified an improvement compared to control.^6^, ^15^, ^25^, ^29^, ^30^, ^40^ Similarly to effects on insulin levels, evidence on insulin resistance in the IF group compared to *ad libitum* groups was inconclusive. Six reviews reported a significant reduction in insulin resistance,^6^, ^21^, ^29^, ^30^, ^41^, ^42^ four reviews found no difference^13^, ^17^, ^24^, ^28^ and one review had mixed results with an isolated study showing a decrease in insulin sensitivity.^20^

#### IF versus CER

Twelve reviews compared fasting glucose in IF and CER diets.^4^, ^6^, ^11^, ^18^, ^19^, ^25^, ^26^, ^27^, ^32^, ^33^, ^37^, ^38^ These reviews included 212 studies and were conducted on predominantly individuals with overweight or obesity. Across 11 reviews, 17 studies included participants with T2D. The remaining review that included participants with T2D did not specify a count.^26^

Ten reviews found no difference in fasting glucose between IF and CER. One review found mixed results attributed to the heterogeneity of studies.^4^ One review found evidence of significantly lower fasting glucose levels in the IF cohort compared to CER (−0.78 mmol/l vs. −0.47 mmol/l, *p* < 0.05), although this was based on a single study.^27^

The evidence for differences in fasting insulin between the two groups is mixed. Five reviews found no significant differences between the two groups.^19^, ^26^, ^27^, ^32^, ^38^ Ezzati et al.^4^ found inconsistent results across different IF variations. One meta‐analysis found a significant effect of IER compared to CER on insulin levels (WMD = −3.66 pmol/l, 95% confidence interval = −9.12 to −0.19, *p* = 0.041), although only 182 participants were included in this analysis.^18^ No differences were found in HbA1c^4^, ^26^, ^27^, ^32^, ^33^, ^37^, ^38^ and HOMA‐IR^26^, ^32^ between IF and CER.

Overall, the results of IF versus *ad libitum* are varied; therefore, it is hard to draw a robust conclusion regarding the effects of IF on fasting glucose, HbA1c, insulin resistance and insulin levels. Conversely, in IF versus CER, the results are more consistent in showing no difference between the two groups. It is important to highlight that most studies were undertaken in populations without T2D and therefore significant changes in these indices may not be expected.

### Lipids

#### IF versus *ad libitum*

Change in lipids (TC, TG, LDL‐C and HDL‐C) was only a secondary outcome measure in many of the reviews, with mixed results. TC levels varied between reviews, with some showing a significant reduction in TC in IF diets compared to an *ad libitum* diet. However, other reviews reported an increase in TC or no difference. Similarly, the effects of IF on TG levels and LDL‐C are mixed, with some reviews finding a reduction, others no change and some an increase. Eight reviews found an increase in either LDL‐C or TG, which is potentially concerning given the risk of dyslipidaemia in people with overweight or obesity.^7^, ^13^, ^15^, ^20^, ^28^, ^35^, ^40^, ^42^

Finally, of the 14 reviews observing HDL levels, 11 showed no effect on these during IF. Two reviews found a possible reduction in HDL following IF. They were both conducted in individuals with T2D or another metabolic disorder.^28^, ^30^ One review identified a study that found a temporary rise in morning HDL following an overnight fast, although this result should be interpreted with caution because of the study's small sample size (*n* = 11).^43^

#### IF versus CER

Nine reviews compared lipid profiles between IF and CER and found no significant difference between the two in any of the parameters.^4^, ^5^, ^6^, ^11^, ^18^, ^19^, ^26^, ^32^, ^38^ There was one exception where one review found CER to be superior to IF with respect to reducing total cholesterol and triglycerides, but equivalent with respect to reducing LDL.^44^

Overall, no substantial conclusions can be drawn as a result of the inconclusive evidence on the outcomes of IF diets on lipid profiles. This could be because of inadequate power in the underlying primary studies, resulting in the inability to detect any difference. However, CER and IF diets appear to have a similar effect on lipid profiles.

### Ramadan

Nine reviews described the effects of RDIF and found that weight was significantly reduced during this period (ranging from −1.02 kg and −1.34 kg). Three reviews found BMI reduced during Ramadan.^45^, ^46^, ^47^ However, some reviews also highlighted that weight significantly increased after follow‐up once fasting ended, resulting in an overall non‐significant decrease in weight.^45^, ^46^, ^48^ Weight loss was also seen more predominantly in those with overweight or obesity pre‐Ramadan.^45^, ^46^ Fat mass was shown to significantly decrease during Ramadan; however, this also became non‐significant after follow‐up.^46^

Several reviews have assessed the impact of RDIF on metabolic parameters with variable results. Jahrami et al.^49^ suggested that RDIF decreased TG and increased HDL‐C. Another review concurred with an improvement in lipoprotein parameters, particularly HDL‐C.^50^ Two reviews stated that definitive answers were lacking on the impact of RDIF on glucometabolic and lipoprotein markers.^47^, ^51^

Overall, RDIF causes reductions in weight, fat mass and BMI. However, this is not maintained, such that, although Ramadan may offer positive metabolic effects in the short term, strategies are needed to ensure maintenance after this period.

## DISCUSSION

Overall, IF is more likely to lead to significant weight loss and a decrease in BMI compared to an *ad libitum* diet. Weight loss and fat loss are more pronounced in those with overweight or obesity compared to normal weight controls. This rapid review evidences that IF and CER have comparable weight loss results; however, new evidence is emerging that in some cases IF may be more effective.^52^ Therefore, IF could be recommended by practitioners as an alternative diet strategy for those who find CER difficult to maintain. Most studies found some reduction in fat mass and none found an increase; therefore, those wishing to reduce fat mass could undertake IF. Notably, a reduction in adiposity did not directly translate into an improvement in metabolic blood makers, with no clear effect of IF on lipid profiles and insulin levels, similar to a lack of clear effect of CER diet on these parameters. In all cases, initiation of IF needs to be discussed with a healthcare professional to provide a safe and supported approach to dietary intervention, ideally by a registered dietician.

The mechanism through which IF results in beneficial effects remains unclear. It is proposed that it is not only through a reduction in caloric intake, but also through the alignment with the circadian rhythm. Previous data suggest that disruption of the circadian rhythm is associated with the development of metabolic diseases and that humans (as well as other organisms) have evolved to optimise physiological processes by aligning with an endogenous circadian clock.^53^ Interestingly, RDIF directly opposes the circadian rhythm as fasting occurs during daytime hours.

Nevertheless, Ramadan fasting can lead to reduced weight, BMI and fat mass; however, these effects may not be sustained after Ramadan finishes. Direct comparison of RDIF with other IF diets is not possible for several reasons. First, the duration of fasting changes every few days on account of the lunar calendar. Second, Ramadan is undertaken worldwide in different countries, climates and seasons, which also impacts the duration of the fast. Finally, given the religious significance of Ramadan, all of the studies are observational and many do not have controls. Consequently, many confounders are not accounted for.

Many of the reviews included in this overview had several limitations. Few reviews examined outcomes beyond 6 months. Moreover, the reviews included in the present study examined different regimes of IF and did not compare different types of IF. Some reviews included both randomised and non‐randomised studies, and thus the effect of confounders could not be eliminated. The heterogeneity of interventions precluded meta‐analysis for most systematic reviews. Populations studied varied, with some reviews looking at healthy adults and some examining obese people living with T2D, which could explain varying outcomes between the reviews. No reviews commented on the potential of metabolic derangement of acute swing in metabolic indices that could follow if an individual alternated between IF and excessive food intake.

Future research should focus on providing long‐term data on metabolic effects and their maintenance. These studies should be accompanied by an analysis of subgroup differences such as gender, age, ethnicity and distinct metabolic diseases. Furthermore, data on adherence, tolerance, dropout rates and sustainability of IF is needed.

### Implications for public health and clinical practice

The worldwide obesity epidemic necessitates identifying effective dietary strategies. Many individuals find the current gold standard CER hard to maintain and fit around daily life. IF might be more favourable because of its emphasis on a restricting eating window and not calories. IF has similar, if not superior, metabolic effects to that of CER in the short‐term (under 6 months)^52^; therefore, when recommending dietary regimes to patients, IF could be offered as an alternative method for those who struggle to maintain CER. Evidence is needed for the long‐term effects of IF.

## AUTHOR CONTRIBUTIONS

All authors contributed to conception and design as part of the Warwick Obesity Network, drafted or revised article and approval final version of the manuscript. ML: methodology, literature search, analysis, writing of original draft and editing. AC: literature search, initial analysis, writing of original draft. PC: conceptualisation, methodology, analysis, writing of original draft, editing. TB: conceptualisation, writing of original draft, editing. TvR: conceptualisation, writing of original draft, editing, funding acquisition. OO: conceptualisation, methodology, writing of original draft, editing. SA: writing: review and editing. PH: conceptualisation, methodology, literature search, writing of original draft, editing, supervision.

## CONFLICTS OF INTEREST STATEMENT

The authors declare that there are no conflicts of interest.

### PEER REVIEW

The peer review history for this article is available at https://www.webofscience.com/api/gateway/wos/peer-review/10.1111/jhn.13253.

## Data Availability

Data sharing is not applicable to this article because no new data were created or analysed in this study.
